# Greater efficacy of intracavitary infusion of bevacizumab compared to traditional local treatments for patients with malignant cavity serous effusion

**DOI:** 10.18632/oncotarget.13064

**Published:** 2016-11-03

**Authors:** Dawei Chen, Xinyu Song, Fang Shi, Hui Zhu, Haiyong Wang, Nasha Zhang, Yan Zhang, Li Kong, Jinming Yu

**Affiliations:** ^1^ Weifang Medical University, Weifang, China; ^2^ Department of Radiotherapy, Shandong Cancer Hospital Affiliated to Shandong University, Jinan, China; ^3^ School of Medicine and Life Sciences, University of Jinan – Shandong Academy of Medical Sciences, Jinan, China

**Keywords:** malignant serous cavity effusion, intracavitary, bevacizumab, chemotherapy, biological response modifiers

## Abstract

Intracavitary infusion of bevacizumab is one effective treatment for malignant serous cavity effusion (MSCE). In this study, we retrospectively evaluated the efficacy of local treatments in 996 advanced cancer patients with MSCE who received paracentesis and intracavitary bevacizumab, or chemotherapy, biological response modifiers, or simple puncture to drain the effusion. The median progression-free survival (PFS) time in patients treated with bevacizumab was 189 days (range, 13-522 days), which was longer than in patients who received one of the other three treatments (*p* < 0.05). Subgroup analysis revealed that intracavitary infusion of bevacizumab was advantageous for patients with malignant pleural, pericardial, or peritoneal effusions. The median PFS in patients receiving intracavitary bevacizumab did not significantly differ from that of patients receiving a combination of intracavitary and intravenous bevacizumab. Thus the efficacy did not depend on whether patients received intravenous bevacizumab. Only mild related adverse events were observed in all cases, and they did not differ between groups. Proteinuria (severity grade < 3) was most likely to occur in patients who received a combination of intracavitary and intravenous bevacizumab, but no obvious symptoms were observed. Thus, intracavitary infusion of bevacizumab was effective for controlling MSCE without apparent toxicity.

## INTRODUCTION

Malignant serous cavity effusion (MSCE), which includes malignant pleural, pericardial, and peritoneal effusions, is the most common complication of advanced cancer [1]. MSCE is frequently observed when anticancer therapy is no longer feasible or available [2]. It most commonly occurs in patients with advanced lung, breast, and ovarian cancer [3, 4], and results in serious symptoms such as dyspnea, pain, vomiting, and fatigue. Because MSCE affects survival rates and is correlated with a poor prognosis [3, 5], it is essential to develop effective treatments. Symptomatic MSCE is typically treated with paracentesis and intracavitary infusion of chemotherapy, biological response modifiers, or simple puncture to drain the effusion. However, the treatment efficacy is unsatisfactory [6, 7].

Vascular endothelial growth factor (VEGF) plays an important role in the development of MSCE [8, 9, 10, 11, 12]. Interestingly, several recent studies have indicated that bevacizumab, a VEGF inhibitor, is effective in preventing MSCE [10, 12, 13]. Kitamura et al. performed a retrospective study of the use of bevacizumab to control MSCE. They determined that intravenous administration of bevacizumab was effective for the management of malignant pleural effusion (MPE) in non-squamous non-small cell lung cancer (NSCLC) patients [14]. Similar prospective studies of the efficacy of intracavitary bevacizumab for the treatment of MSCE have demonstrated satisfactory response rates in patients [14, 15].

The optimal therapeutic approach for MSCE treatment in patients with advanced cancer has not yet been determined. Although studies have reported that intracavitary infusion of bevacizumab improved symptoms in cancer patients with MSCE [6, 14, 15,16, 17, 18], few parallel studies have been performed to compare intracavitary bevacizumab treatment with traditional local treatments for MSCE. Additionally, there is no direct evidence that intracavitary bevacizumab is better than the traditional treatments. Intravenous administration of bevacizumab in combination with systemic chemotherapy was effective for the treatment of MPE in NSCLC patients [7, 14]. Here, we assessed the efficacy and toxicity of intracavitary infusion of bevacizumab compared to several chemotherapeutic agents, biological response modifiers, and simple puncture to drain the effusion.

## MATERIALS AND METHODS

### Patients

A total of 1,371 cancer patients with MSCE were treated at Shandong Cancer Hospital and Institute between August 2009 and February 2015. All patients received systemic chemotherapy for primary tumors and local treatment for MSCE. We excluded 310 patients because they received various combinations of the four treatments assessed in our study. In addition, 47 patients were excluded because they underwent pleurodesis prior to the removal of the indwelling cavitary catheter. Eight patients were excluded because an examination did not indicate cavity effusion in a timely manner. Twenty patients were excluded because survival data was not available and five because the systemic therapy regimen was not available. A total of 996 advanced cancer patients with MSCE were included in our analysis. Patient data including the type of cancer and effusion are shown in Table 1. The patients were classified into subgroups based on the local treatment regimen for MSCE: Group 1, paracentesis and intracavitary bevacizumab; Group 2, paracentesis and intracavitary chemotherapy; Group 3, paracentesis and intracavitary biological response modifiers; and Group 4, simple puncture to drain the effusion (Table 1). Patients who received systemic therapy for the treatment of primary tumors were divided into two groups based on whether they received intravenous bevacizumab. Finally, patients were classified into the following subgroups based on the type of effusion: the MPE group, malignant ascites (MA) group, and malignant pericardial effusion (MPCE) group. This study was approved by the Ethics Committee of Shandong Cancer Hospital and Institute (Jinan, Shandong, China).

**Table 1:** Characteristics of the patients and treatments selected [*n* (%)]

	Overall (*N* = 996)	Group 1 (*N* = 72)	Group 2 (*N* =530)	Group 3 (*N* = 298)	Group 4 (*N* = 96)	*p* Value
Age (years)						
>65	695 (69.78)	51 (70.83)	350(66.04)	218 (73.15)	66 (68.75)	
≤65	301 (31.22)	21 (29.17)	180 (33.96)	80 (26.85)	30 (31.25)	0.199
Sex						
Male	501 (50.30)	38 (52.78)	284 (53.58)	132 (44.30)	47 (48.96)	
Female	495 (49.70)	34 (47.22)	246 (46.42)	166 (55.70)	49 (51.04)	0.078
ECOG						
0–2	681 (68.37)	57 (79.17)	367 (69.25)	206 (69.13)	61 (63.54)	
≥2	315 (31.63)	15 (20.83)	163 (30.75)	92 (30.87)	35 (36.46)	0.187
Clinical stage						
Stage III	346(34.74)	18(25.00)	165(31.13)	90(30.20)	36(37.5)	
Stage IV	650(65.26)	54(75.00)	365(68.87)	208(69.80)	60(62.5)	0.362
Type of tumor						
Lung cancer	520 (52.2)	28 (38.89)	221 (41.7)	203 (68.12)	48 (50.00)	
Ovarian cancer	79 (7.93)	9 (12.5)	74 (13.96)	6 (2.01)	10 (10.41)	
Breast cancer	60 (6.02)	11 (15.28)	40 (7.55)	7 (2.35)	2 (2.04)	
CRC	49 (4.92)	9 (12.5)	49 (9.24)	15 (5.03)	3 (3.06)	
Cervical cancer	54 (5.42)	1 (1.38)	45 (8.49)	5 (1.68)	8 (8.33)	
Gastric cancer	51 (5.12)	6 (8.33)	40 (7.55)	5 (1.68)	3 (3.12)	
UPSC	69 (6.93)	5 (6.94)	40 (7.55)	20 (3.77)	4 (4.16)	
Lymphoma	59 (5.92)	2 (2.78)	20 (3.77)	37 (6.98)	17 (17.70)	
Others	55 (5.52)	1 (1.38)	1 (0.19)	0	3 (3.12)	<0.01
Type of effusion						
MPE	574 (57.63)	50 (69.44)	250 (47.17)	216 (72.48)	58 (60.42)	
MA	297 (29.82)	12 (16.67)	211 (39.81)	64 (21.48)	10 (10.42)	
MPCE	125 (12.55)	10 (13.89)	69 (13.02)	18 (6.04)	28 (29.16)	<0.01
Intravenous Bev						
Yes	363 (36.44)	37 (51.39)	204(38.49)	95(31.88)	27(28.13)	
No	633 (63.56)	35 (48.61)	326(61.51)	203(68.12)	69(71.87)	<0.01

Abbreviations: Group 1, intracavitary bevacizumab; Group 2, intracavitary chemotherapy; Group 3, intracavitary biological response modifiers; Group 4, simple puncture to drain the effusion; CRC, colorectal and rectum cancer; UPSC, cancer of unknown primary site.

### Bevacizumab treatment and dosage

All patients required ultrasound-guided pleural, peritoneal, or pericardial catheterization. Each patient underwent drainage to remove as much of the MSCE as possible. Intracavity administration of the therapeutic agents was then performed. Bevacizumab was infused at a dose of 100 or 200 mg diluted in 50 mL of physiological saline. This treatment was repeated every week until a response was observed. The chemotherapeutic agents we analyzed were cisplatin, 5-fluorouracil, and bleomycin, and the biological response modifiers were lentinan and IL-2R. Patients were required to carefully turn over every 10 min to ensure sufficient absorption of the therapeutic agents in the serous cavity.

### Data collection and evaluation criteria

Clinicopathological data were collected for all patients, which included treatment response. Short-term therapeutic efficacy was evaluated as previously described [7, 15, 17] and according to the Response Evaluation Criteria in Solid Tumors version 1.1. Short-term efficacy was classified as either complete response (CR; effusion and symptoms disappeared and the patient was stable for > 8 weeks), partial response (PR; the size of the effusion was reduced by 50%, symptoms improved, and no subsequent growth in the effusion was observed over an 8-week period); stable disease (SD; the effusion size was reduced by < 50% or remained unchanged; and progressive disease (PD; the effusion size increased). The objective response rate (ORR) involved the assessment of CR and PR, and the effusion control rate (ECR) involved the assessment of CR, PR, and SD. Progression-free survival (PFS) was defined as the interval between the initiation of local treatment and the time of either effusion progression or death.

Safety was evaluated based on adverse events (AEs), relevant laboratory findings, and vital signs. Safety reporting was performed according to the relevant ICH Good Clinical Practice guidelines. The National Cancer Institute Common Terminology Criteria for Adverse Events version 4.0 were used to grade AEs.

### Statistical analysis

All statistical analyses were performed using the Statistical Package for the Social Sciences version 17.0 software (SPSS Inc., Chicago, IL, USA). Data and short-term efficacy were analyzed using chi-square and *t*-tests. Independent prognostic factors for patient outcome were identified using a Cox regression model. PFS was analyzed using the Kaplan-Meier method and differences were evaluated using log-rank tests. Two-sided *P* values < 0.05 were considered statistically significant.

## RESULTS

### Patient characteristics

A total of 996 patients (501 men and 495 women, median age = 69 years) were enrolled in our study. All patients received local treatment for MSCE and systemic chemotherapy for the treatment of primary tumors. There were 72 patients who underwent paracentesis and received intracavitary bevacizumab, 530 who underwent paracentesis and received intracavitary chemotherapy, 298 who underwent paracentesis and received intracavitary biological response modifiers, and 96 who underwent simple puncture to drain the effusion. Additionally, there were 363 patients who received systemic chemotherapy with intravenous bevacizumab and 633 who received systemic chemotherapy without intravenous bevacizumab. A total of 574 patients had MPE, 297 had MA, and 125 had MPCE. All patients were followed-up for a median duration of 11.2 months. The final follow-up session occurred in February 2016. During the follow-up period, 954 patients either had a recurrence of the effusion recurrence or died of cancer. The patient characteristics are shown in Table 1. No significant differences were observed between the treatment groups.

We assessed the prognostic value of various patient clinicopathological characteristics for PFS (Table 2). Univariate analysis revealed that the ECOG score (*p* = 0.031), type of effusion (*p* = 0.036), and treatment with intravenous bevacizumab (*p <* 0.01) were associated with effusion control. Multivariate analysis revealed that the type of effusion (*p* = 0.015) and treatment with intravenous bevacizumab (*p <* 0.01) were independent prognostic factors for PFS.

**Table 2:** Factors associated with PFS in univariate and multivariate analyses

	Univariate analysis			Multivariate analysis		
	HR	95% CI	*p*-value	HR	95% CI	*p*-value
Age	0.983	0.952-1.017	0.362			
Sex	1.115	0.773-1.869	0.69			
ECOG score*	0.763	0.591-0.975	0.031*	1.370	0.757-2.457	0.296
Clinical stage	1.284	0.896-1.828	0.16			
Type of tumor	1.426	0.673-1.869	0.66			
Type of effusion*	0.683	0.650-1.871	0.036*	0.491	0.759-1.466	0.015*
Intravenous Bev*	0.674	1.767-2.617	<0.01*	0.768	0.748-1.617	<0.01*

Abbreviationss: * Statistically significant; PFS, progression-free survival; HR, hazards ratio; CI, confidence interval.

### Treatment efficacy in all 996 patients

**Figure 1:**
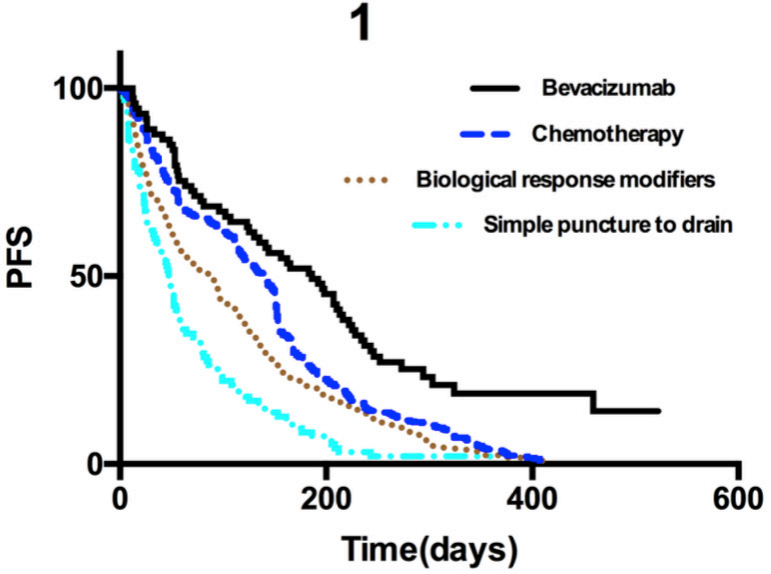
Kaplan-Meier curves for progression-free survival in all 996 patients *p < 0.05 for bevacizumab compared to chemotherapy; **p < 0.05 for bevacizumab compared to biological response modifiers; ***p < 0.05 for bevacizumab compared to simple puncture to drain the effusion.

The PFS data for all 996 patients were stratified according to the different treatments and analyzed using Kaplan-Meier curves and log-rank tests (Figure 1). Analysis of short-term and long-term efficacy indicated that intracavitary infusion of bevacizumab was advantageous. We analyzed short-term efficacy and determined that the ECR and objective response rate (ORR) of the patients who received intracavitary infusion of bevacizumab were 87.5% and 77.78%, respectively, which were higher than those of the patients who received one of the other three treatments (*p* < 0.05). Analysis of long-term efficacy revealed that the median PFS of all 996 patients was 112 days (range, 1-522 days), and that the median PFS in patients who received intracavitary bevacizumab, chemotherapy, biological response modifiers, or simple puncture to drain the effusion was 186 days (range, 12-522 days), 141 days (range, 1-412 days), 87 days (range, 1-413 days), and 47 days (range, 3-287 days), respectively. The median PFS of patients who received intracavitary bevacizumab was higher than that of patients who received one of the other three treatments (*p <* 0.05)

Multivariate analysis revealed that the type of effusion and treatment with intravenous bevacizumab were independent prognostic factors for PFS. Meanwhile, in characteristics of the patients, the distribution of type of effusion and intravenous bevacizumab were imbalanced. Considering this, we took subgroup analysis to assess the efficacy in patients with different type of effusion and the efficacy in patients who did or did not receive intravenous administration of bevacizumab.

### Efficacy in patients with malignant pleural effusion, malignant ascites, or malignant pericardial effusion

**Figure 2:**
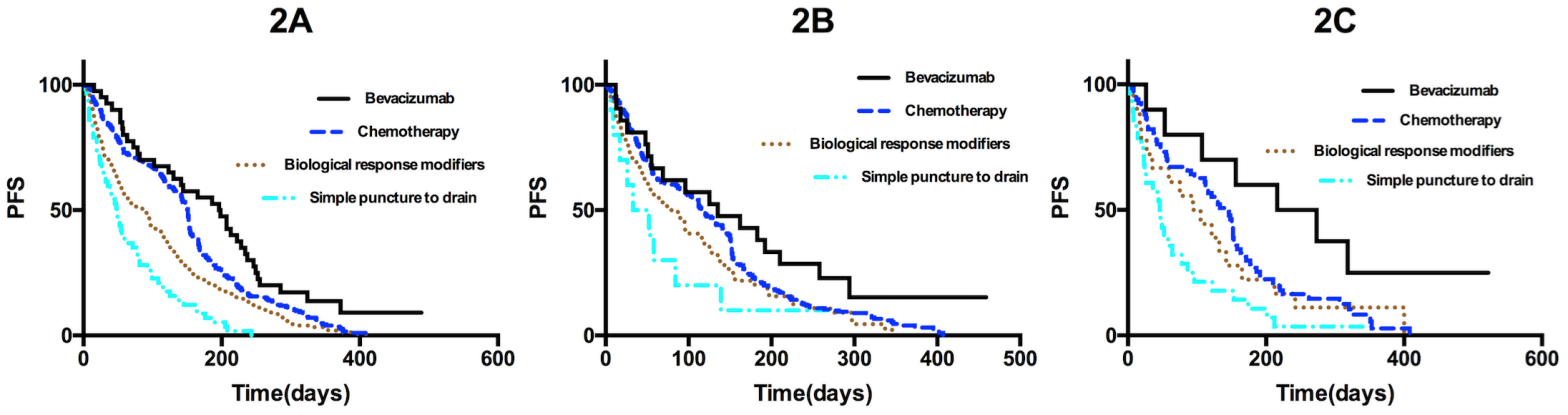
A. Kaplan-Meier curves for progression-free survival in all 574 patients with MPE *p < 0.05 for bevacizumab compared to chemotherapy; **p < 0.05 for bevacizumab compared to biological response modifiers; ***p < 0.05 for bevacizumab compared to simple puncture to drain the effusion. **B**. Kaplan-Meier curves for progression-free survival in all 297 patients with MA. *p < 0.05 for bevacizumab compared to chemotherapy; **p < 0.05 for bevacizumab compared to biological response modifiers; ***p < 0.05 for bevacizumab compared to simple puncture to drain the effusion. **C**. Kaplan-Meier curves for progression-free survival in all 125 patients with MPCE. *p < 0.05 for bevacizumab compared to chemotherapy; **p < 0.05 for bevacizumab compared to biological response modifiers; ***p < 0.05 for bevacizumab compared to simple puncture to drain the effusion.

The 996 patients were divided into three groups according to the type of effusion: 574 patients had MPE, 297 had MA, and 125 had MPCE. The results for the efficacy of intracavitary bevacizumab are shown in Table 2. Analysis of short-term efficacy indicated that patients who received intracavitary bevacizumab had greater ECR and ORR, regardless of the type of effusion. The median PFS of patients with MPE, MA, and MPCE was 115 days (range, 3-489 days), 101.5 days (range, 1-459 days), and 112 days (range, 3-522 days), respectively. The median PFS of patients who received intracavitary bevacizumab was higher than that of patients who received the other three treatments (*p* < 0.05, Figure 2A-2C; respectively).

### Efficacy in patients who did or did not receive intravenous administration of bevacizumab

**Figure 3:**
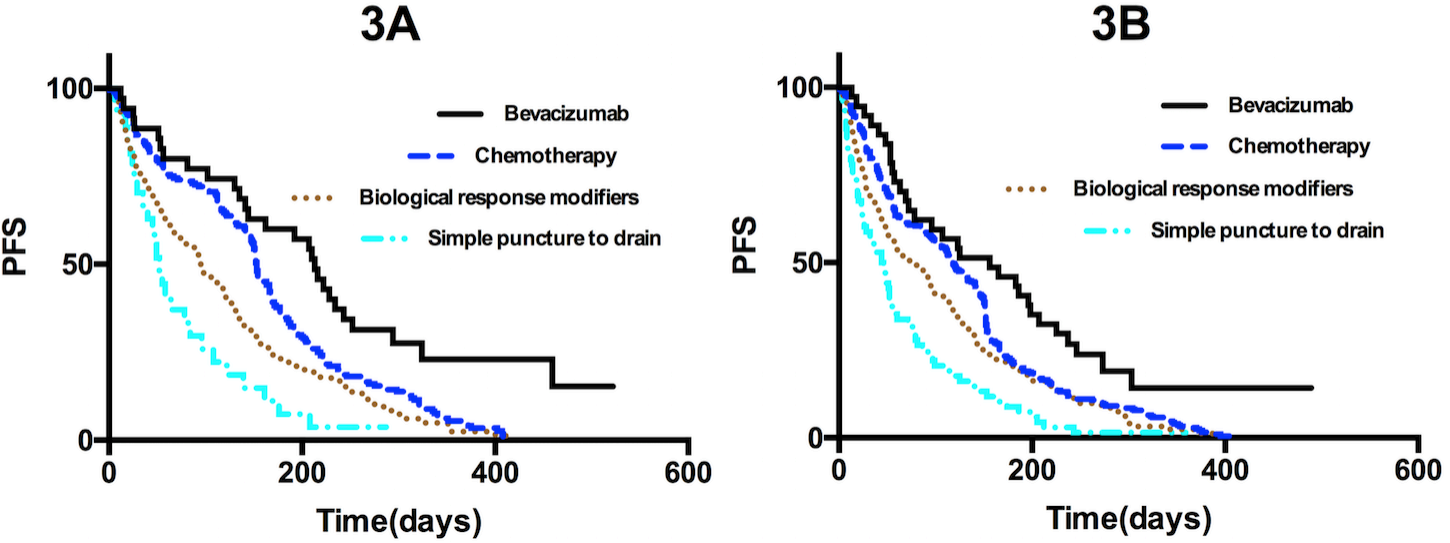
A. Kaplan-Meier curves for progression-free survival in all 363 patients who received systemic therapy with intravenous administration of bevacizumab *p < 0.05 for bevacizumab compared to chemotherapy; **p < 0.05 for bevacizumab compared to biological response modifiers; ***p < 0.05 for bevacizumab compared to simple puncture to drain the effusion. **B**. Kaplan-Meier curves for progression-free survival in the 633 patients who received systemic therapy without intravenous administration of bevacizumab. *p < 0.05 for bevacizumab compared to chemotherapy; **p < 0.05 for bevacizumab compared to biological response modifiers; ***p < 0.05 for bevacizumab compared to simple puncture to drain the effusion.

A total of 363 patients received systemic therapy with intravenous administration of bevacizumab and 633 patients received systemic therapy without intravenous administration of bevacizumab. The short-term efficacy was significantly better in patients who received intracavitary administration of bevacizumab compared to those who received traditional local treatments (*p <* 0.05) (Table 2). The two groups showed significant differences in long-term efficacy. The median PFS was higher among the 363 patients who received intravenous administration of bevacizumab than among those who received biological response modifiers or simple puncture to drain the effusion (*p* < 0.05), but did not differ from that of patients who received intracavitary administration of chemotherapy (*p* = 0.411, Figure 3A). The median PFS after intracavitary administration of bevacizumab was significantly higher among the 633 patients who did not receive intravenous administration of bevacizumab compared to patients who received one of the other three treatments (*p* < 0.01, Figure 3B).

### Adverse events

There were three types of AEs: grade ≥ 3 AEs, specific AEs, and bevacizumab-related AEs. The incidence of all AEs is shown in Table 3. Major AEs including gastrointestinal reactions and hematologic toxicities occurred in all groups. However, these symptoms were typical side effects of systemic chemotherapy and not likely due to intracavitary administration of bevacizumab. The incidence of the most common complications associated with bevacizumab such as hypertension, thrombus, perforations, and bleeding, did not significantly differ between the four groups. However, the incidence of proteinuria was higher in the intracavitary bevacizumab group. We found that 12 of 72 patients had proteinuria (16.67%). Further analysis showed that 9 of the 12 patients who received intracavitary administration of bevacizumab also received intravenous administration of bevacizumab. Although proteinuria is a common complication of bevacizumab, there is no clear evidence of increased AEs after intracavitary administration of bevacizumab. In addition, the grade of severity was < 3 among all patients with proteinuria and only one patient required continuous treatment to maintain kidney function.

**Table 3:** Comparison of responses to treatments in all the patients, patients with different effusion type, and patients who received or did not receive intravenous administration of Bev [n (%)] in different groups [days]

		Group 1	Group 2	Group 3	Group 4	*p* Value
Overall		72	530	298	96	
CR		13 (18.05)	48 (9.05)	11 (3.69)	0	*<0.01*
PR		43 (59.72)	328 (61.89)	151 (50.67)	37 (38.54)	*<0.01*
SD		7 (9.72)	32 (6.04)	25 (8.39)	7 (7.29)	0.493
ECR		63 (87.5)	408 (76.98)	187 (62.75)	44 (45.83)	*<0.01*
ORR		56 (77.78)	376 (70.94)	162 (54.36)	37 (38.54)	*<0.01*
Different effusion type						
MPE (*n* = 574)	ECR	45 (90.00)	206 (82.40)	146 (67.59)	27 (46.55)	*<0.01*
	ORR	41 (82.00)	191 (76.40)	128 (59.26)	23 (36.65)	*<0.01*
MA (*n* = 297)	ECR	10 (83.33)	145 (68.72)	30 (46.87)	4 (40.00)	*<0.01*
	ORR	9 (75.00)	134 (63.51)	25 (39.06)	3 (30.00)	*<0.01*
MPCE (*n* = 125)	ECR	8 (80.00)	57 (82.61)	11 (61.11)	13 (46.43)	*<0.01*
	ORR	6 (60.00)	51 (73.91)	9 (50.00)	10 (35.71)	*<0.01*
Intravenous Bev						
Yes	ECR	34 (91.89)	168 (82.35)	61 (64.21)	20 (54.05)	*<0.01*
	ORR	31 (83.78)	155 (75.98)	55 (57.89)	18 (48.64)	*<0.01*
No	ECR	29 (82.86)	240 (73.62)	126 (62.07)	24 (40.68)	*<0.01*
	ORR	25 (71.42)	221 (67.79)	107 (52.70)	19 (32.20)	*<0.01*

Abbreviations: ORR, objective response rate; ECR, disease control rate; CR, complete response; PR, partial response; SD, stable disease; PD, progressive disease; mPFS, median progression-free survival; Bev, bevacizumab.

**Table 4:** Comparison of adverse events among the treatment groups [*n* (%)]

	Group 1	Group 2	Group 3	Group 4	*p* Value
**Grade ≥ 3**	12 (16.66)	101 (19.05)	39 (13.09)	10 (10.42)	0.054
**Specific AEs**					
Neutropenia	18 (25.00)	159 (30.00)	81 (27.18)	24 (25.00)	0.131
Anemia	6 (8.33)	43 (8.11)	29 (9.73)	6 (6.25)	0.726
Thrombocytopenia	6 (8.33)	58 (10.94)	18 (6.04)	9 (9.37)	0.133
Febrile neutropenia	5 (4.25)	27 (5.09)	21 (7.04)	6 (6.25)	0.686
Leukopenia	10 (13.89)	80 (15.09)	33 (11.07)	12 (12.5)	0.434
Hypertension	3 (4.17)	27 (5.09)	9 (3.02)	3 (3.12)	0.503
Vomiting	4 (5.56)	37 (6.98)	18 (6.04)	5 (5.21)	0.880
Diarrhea	1 (1.39)	16 (3.02)	12 (4.02)	3 (3.12)	0.689
Infection	0	11 (2.07)	9 (3.02)	2 (2.08)	0.460
**AEs related with Bev**	28 (38.89)	238 (44.90)	125 (41.94)	37 (38.54)	0.533
Proteinuria*	12 (16.67)	32 (6.03)	15 (5.03)	4 (4.17)	<0.01*
Hypertension	3 (4.17)	27 (5.09)	9 (3.02)	3 (3.12)	0.503
Thrombus	2 (2.78)	1 (0.19)	2 (0.67)	1 (1.04)	0.060
GI perforations	1 (1.34)	3 (0.56)	2 (0.67)	0	0.715
Bleeding	2 (2.78)	3 (0.56)	2 (0.67)	0	0.153

Abbreviations: AEs, adverse events; GI, gastrointestinal.

## DISCUSSION

Fluid is normally produced in the pleural, pericardial, and abdominal cavities of healthy individuals. A balance of lymphatic production and absorption maintains equilibrium [19]. Pathological processes can accelerate or block effusion production, which leads to the accumulation of excess fluid. MSCE is a frequent and severe complication of advanced cancer. It can occur through two mechanisms: (1) tumor cell secretion of VEGF and other cytokines that increase capillary permeability and promote angiogenesis, and (2) occlusion of the lymphatic conduit by cancer cells, which can increase the hydrostatic pressure, disrupt the flow of lymphatic effusions, reduce water and protein absorption, and lead to fluid retention in the serous cavity [20, 21]. MSCE often results in complications that can affect patient quality of life and shorten survival times [22].

Both local and systemic therapies have been used to treat MSCE. Local treatments include paracentesis and intracavitary infusion of chemotherapy, biological response modifiers, and simple puncture to drain the effusion. Paracentesis and intracavitary infusion of chemotherapy can inhibit tumor progression. Therefore, it is the most common local treatment combination for MSCE. However, it can promote adhesion of the serosal membrane to the cavity. In addition, patients with advanced cancer usually cannot tolerate the AEs associated with systematic chemotherapy. Intracavitary infusion of biological response modifiers is an alternative treatment option for MSCE. Although biological response modifiers have suitable anti-tumor effects, they can also cause AEs and adhesion of the serosal membrane and cavity. Importantly, this type of treatment only relieves symptoms temporarily. Similarly, a simple puncture to drain the effusion typically only offers only temporary relief. It can also lead to a loss of protein and electrolytes, and increase the chance of infection, errhysis, and other complications. Overall, systemic treatments are essential for patients with MSCE of neoplastic origin. Because most patients receive multiples lines of chemotherapy, they may not tolerate an increase in pain and toxicity. Thus, neither traditional local treatments nor systemic therapy can achieve satisfactory patient outcomes.

Elevated VEGF expression, increased vascular permeability, and angiogenesis underlie the development of MSCE [12]. Angiogenesis was shown to shown to contribute to the production of MSCE [23], and overexpression of VEGF, a major angiogenic factor, has been frequently observed in tumor cells [24]. VEGF can cause tumor vessels to become disorganized, leaky, and tortuous. It also promotes the production of MSCE by perturbing the balance between fluid production and lymphatic absorption [23, 25].

Bevacizumab is a recombinant, humanized monoclonal anti-VEGF antibody that consists of the antigen-binding complementarity-determining regions of a murine antibody. It blocks binding of human VEGF to its receptors [26]. Bevacizumab was shown to inhibit the growth of 13 different types of malignant cells, and reduced the density, diameter, and permeability of vessels [27]. Several studies have demonstrated that intracavitary administration of bevacizumab was a safe and effective therapeutic approach for controlling MSCE [13, 26, 28–30].

Intracavitary administration of bevacizumab has yielded favorable outcomes in patients with MPE and ascites. Kazuhiro et al. [14] reported an ORR of 45.5% and median PFS time of 312 days in NSCLC patients with MPE. There was no evidence of treatment-related toxicities. In addition, Chen et al. [6] reported an ORR of 65.21%, ECR of 86.96%, and median PFS time of 6 months in mesothelioma patients with MPE. All of the patients in the study tolerated the treatment. Although the local application of bevacizumab for MSCE has been shown to be safe and effective, no previous studies have compared bevacizumab to other traditional local treatments in advanced cancer patients with MSCE. Many patients received intracavitary administration of bevacizumab while also receiving systemic therapies including intravenous administration of bevacizumab. Consistent with previous studies [7, 14], intravenous administration of bevacizumab was highly effective for the management of MPE. Thus, we hypothesize that intravenous administration of bevacizumab could be used to treat both MPCE and MA. However, no previous studies involving intracavitary infusion of bevacizumab have considered the effects of intravenous bevacizumab.

Our study is the first to directly compare intracavitary bevacizumab therapy with other traditional local treatments for MSCE. We confirmed that intracavitary administration of bevacizumab was safe and effective for the management for MSCE. Several related studies have focused on comparing intracavitary infusion of bevacizumab with cisplatin [6, 7, 16]. In our study, we also assessed treatment with biological response modifiers or simple puncture to drain the effusion. Previous studies only analyzed patients with lung and ovarian cancer [14, 15, 17]. In contrast, we studied patients with various cancers including breast, colorectal, and rectal cancer. Our study has two major strengths. First, we analyzed three groups of patients (MPE, MA, and MPCE) because the prognosis of patients with these different types of effusions is different. Interestingly, we found that intracavitary infusion of bevacizumab was more effective than the other three treatments in all groups of patients. Second, we classified patients into subgroups according to whether they received intravenous administration of bevacizumab. We determined that intracavitary infusion of bevacizumab was advantageous regardless of whether patients received intravenous administration of bevacizumab. The median PFS for patients who received intracavitary bevacizumab did not differ from that of patients who received intracavitary chemotherapy without intravenous bevacizumab.

Our study also had several limitations. First, there was inherent bias owing to the retrospective nature of the study. Second, all of the patients were Chinese individuals treated at a single hospital. Therefore, the findings may not be applicable to other populations. A large prospective study is now required to validate our findings.
